# Re-Irradiation With Proton Beam Therapy for Localized Perineural Spread Following Presacral Recurrence in Sigmoid Colon Cancer: A Case Report

**DOI:** 10.7759/cureus.56765

**Published:** 2024-03-23

**Authors:** Masahiko Harada, Takashi Saito, Toshiki Ishida, Yutaro Mori, Hideyuki Sakurai

**Affiliations:** 1 Department of Radiation Oncology, University of Tsukuba, Tsukuba, JPN

**Keywords:** presacral recurrence, perineural spread, re-irradiation, colorectal cancer, proton beam therapy

## Abstract

This report describes the effective management of localized perineural spread (PNS) to the sacral peripheral nerves following a presacral recurrence of colon cancer using proton beam therapy (PBT). The patient, a male in his 60s with a history of sigmoid colon cancer treated with laparoscopic Hartmann's procedure, presented with presacral recurrence two years post-surgery. Radical resection was deemed infeasible, leading to a combined treatment of PBT (75 Gy relative biological effectiveness (RBE) in 25 fractions) and capecitabine. However, three years post-PBT, magnetic resonance imaging revealed swelling of the left S2 nerve with abnormal fluorodeoxyglucose uptake, indicating localized PNS. Re-irradiation with PBT (75 Gy RBE in 25 fractions) was conducted, carefully considering the overlap with the previous PBT field and aiming to minimize dosage to adjacent organs. At 1.5 years post-reirradiation, the patient remained free of recurrence. This case underscores the potential efficacy of PBT and emphasizes the need for further research to assess its broader applicability in comparable situations.

## Introduction

Approximately 10% of cases of rectal cancer have local recurrence postoperatively, with about half of these occurring anterior to the sacrum [1,2]. In recurrence anterior to the sacrum, the proximity of recurrent lesions to the sacrum often complicates re-excision, making radiation therapy a viable option [3]. However, due to the low radiosensitivity of colorectal cancer, there is a need to use strategies such as spacers, particle beam therapy, or stereotactic body radiation therapy (SBRT) to minimize the dose to surrounding organs while delivering a high dose to the tumor [4]. In some cases, there have been reports of perineural spread (PNS) of pelvic cancers to the lumbosacral plexus and sacral peripheral nerves [5]. In presacral recurrence of colorectal cancer, the lesions often abut the sacral nerves, potentially leading to PNS, but there are few reports describing treatment methods for this condition.

Here, we present a case in which high-dose proton beam therapy (PBT) was administered for presacral recurrence of colon cancer. Three years later, localized PNS was observed along the sacral nerve, and subsequent re-irradiation with PBT achieved a complete response for this lesion.

## Case presentation

**Figure 1:**
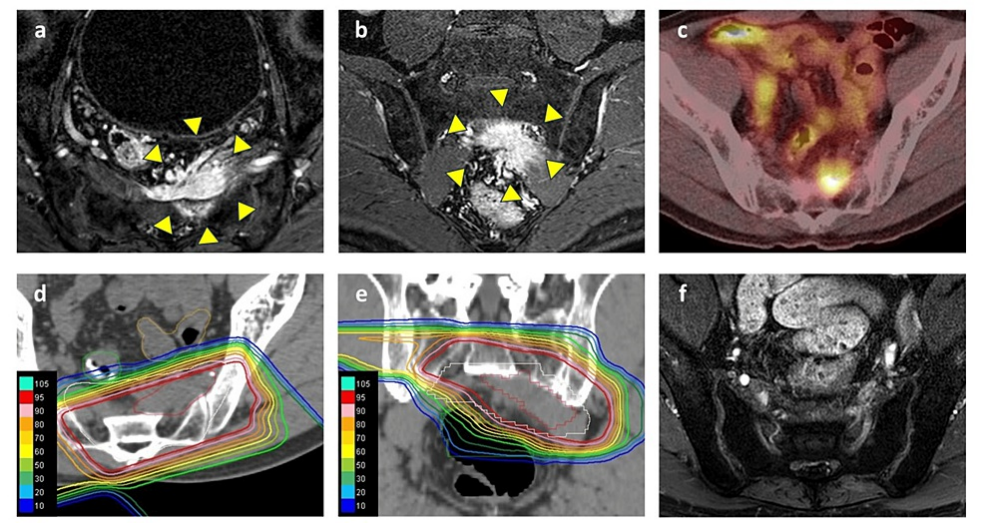
MRI and FDG PET-CT images pre and post-first PBT for presacral recurrence Contrast-enhanced MRI (a: axial, b: coronal), FDG PET-CT (c: axial), and dose distribution of first PBT (d: axial, e: coronal). Post-treatment MRI (f: axial) showed a complete response one year after the first PBT. In the images, the recurrent lesion is indicated by a yellow triangle. The dose lines are color-coded as follows: Red line: 95% isodose line, Yellow line: 75% isodose line, Green line: 50% isodose line, Light blue line: 25% isodose line. FDG PET-CT: 18 F-fluorodeoxyglucose Positron Emission Tomography–Computed Tomography; PBT: Proton Beam Therapy.

A male in his 60s with a history of a laparoscopic Hartmann's procedure for sigmoid colon cancer visited our gastroenterology department following the detection of presacral recurrence on follow-up computed tomography (CT) conducted two years post-surgery. The pathology revealed a 50 mm moderately differentiated adenocarcinoma with serosal invasion. With five out of 17 lymph nodes involved, the staging is classified as pT3N2aM0, according to the 8th edition of the Union for International Cancer Control (UICC) staging system [6]. The patient had no other significant medical history and no history of pre- or postoperative radiation but complained of left buttock pain. The presacral recurrence was located on the level of the second to third sacral bones with infiltration into the left piriformis muscle and showed marked fluorodeoxyglucose (FDG) uptake (standardized uptake value (SUV)max 9.9) on 18 F-fluorodeoxyglucose (FDG) positron emission tomography (PET)-CT (Figures 1a-1c). A multidisciplinary cancer conference concluded that radical resection was not feasible due to the extensive sacral infiltration. As a result, a treatment regimen combining PBT (75 Gy relative biological effectiveness (RBE) in 25 fractions (Figures 1d, 1e)) with concurrent administration of capecitabine was devised. Capecitabine was prescribed twice daily, 3000 mg/day. The detailed methodology for PBT in our facility has been described elsewhere [7]. Follow-up CT or magnetic resonance imaging (MRI) was performed every three months after PBT. After one year, the MRI showed a complete response (Figure 1f), and the symptoms had also improved.

**Figure 2:**
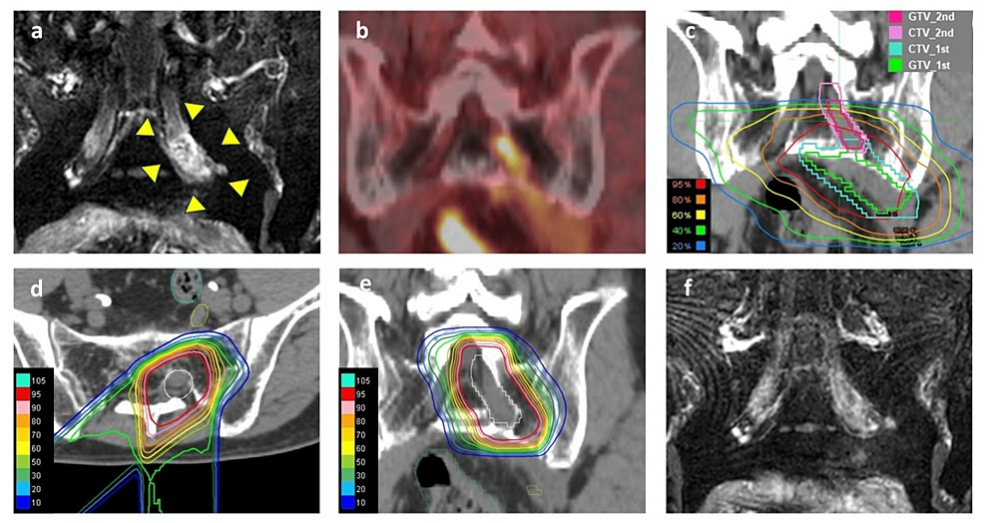
MRI and FDG PET-CT images pre and post-proton beam re-irradiation for localized PNS. Contrast-enhanced MRI (a: coronal), FDG PET-CT (b: coronal), and the relationship of the PNS lesion and first PBT dose. The first gross tumor volume (GTV) is enclosed by the green line. The first clinical target volume (CTV) is enclosed by the blue line. The second GTV is enclosed by a magenta line. The second CTV is enclosed by a pink line (c: coronal). Dose distribution for the proton beam re-irradiation (d: axial, e: coronal). Post-treatment MRI (f: coronal) showed a complete response one year after proton beam re-irradiation. In the images, the recurrent lesion is indicated by a yellow triangle. The dose lines are color-coded as follows: Red line: 95% isodose line, Yellow line: 75% isodose line, Green line: 50% isodose line, Light blue line: 25% isodose line. FDG PET-CT: 18 F-fluorodeoxyglucose Positron Emission Tomography–Computed Tomography

However, two years post-PBT, the patient developed numbness and pain in the left lower limb, with an increase in the carcinoembryonic antigen (CEA) level. MRI did not show any definite indication of recurrence, which suggested the presence of radiation-induced lumbosacral plexopathy (LSP). As a result, oral administration of mirogabalin was started. Despite this treatment, symptoms had worsened at three years post-PBT and MRI showed swelling of the left S2 nerve, a high T2 signal, and contrast enhancement, with corresponding abnormal FDG uptake on FDG PET-CT (SUVmax 9.2) (Figures 2a, 2b). Comparison of the affected left S2 nerve with the PBT field showed that part of the lesion was outside the high-dose area, suggesting localized PNS of colon cancer over radiation-induced LSP (Figure 2c). No further metastases were detected in the CT scans.

**Figure 3:**
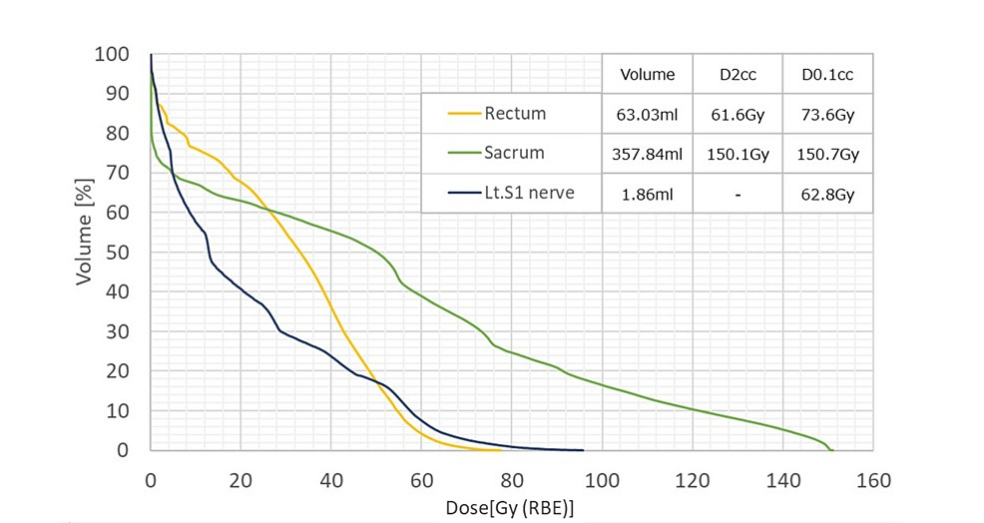
The total dose volume histogram Combined dose of initial and re-irradiation of organs at risk and the parameters of volume D2cc, D0.1cc.

**Figure 4:**
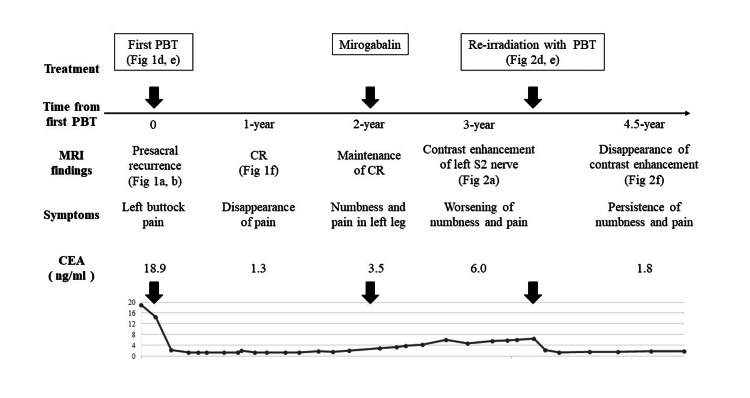
Clinical course depicting key events and the corresponding figure number PBT: Proton Beam Therapy; CEA: Carcinoembryonic Antigen; Fig: Figure/Figures; CR: complete response

A neurological consultation at our hospital revealed sensory impairment and increased reflexes in the posterior region of the left lower leg. A multidisciplinary cancer conference determined that excision or biopsy was not feasible, resulting in the decision to proceed with a re-irradiation with PBT. The re-irradiation with PBT (75 Gy (RBE) in 25 fractions) was administered with consideration of overlap with the previous PBT field and with the aim of minimizing the dosage to surrounding organs such as the left S1 nerve and sacrum bone (Figures 2d, 2e). The total dose-volume histogram (combined dose of initial and re-irradiation) for the organs at risk is shown in Figure 3. At one year post-reirradiation, MRI showed the disappearance of the lesion (Figure 2f), with reduced CEA. At 1.5 years post-reirradiation, the patient remains free of recurrence but continues to have numbness and pain in the left lower limb, necessitating ongoing analgesic medication. The whole clinical course is illustrated in Figure 4.

## Discussion

The occurrence of PNS in colon cancer is rare and there is limited information on its management [5]. This report is the first description of re-irradiation with PBT for localized PNS following presacral recurrence in sigmoid colon cancer.

PNS to the lumbosacral plexus can occur irrespective of the cancer type in pelvic malignancies [5]. Typically, progressive LSP in patients with a history of malignancy prompts diagnosis of PNS. In our case, left lower limb pain developed two years post-initial PBT for presacral recurrence, and progressed over time. The differential diagnosis for progressive LSP with a post-radiation history includes radiation-induced LSP [8], with an increased incidence depending on dose volume, particularly above 60 Gy [9]. Radiation-induced LSP typically shows a T2 high signal and contrast enhancement in the irradiated area on MRI [10], similar to cancer-related LSP [11]. Biopsy is ideal for differentiation, but challenging and risky due to the anatomical location of the sacral nerve [12]. The key differential factor in our case was the relationship between the PBT field and abnormal change at the sacral peripheral nerve. The irradiation dose in the first PBT of the cranial region of the affected left S2 nerve was insufficient for radiation-induced LSP, and the lack of FDG uptake in higher-dosed areas contradicted radiation-induced LSP. The correct cancer-related LSP diagnosis was confirmed by the disappearance of the lesion post-PBT. Although the lesion disappeared, the symptoms continued, making it difficult to definitively rule out radiation-induced LSP. The possibility for the coexistence of both conditions is feasible, however, it was not possible to make further differentiation. Given the low radiosensitivity of colon cancer, administering high-dose PBT is necessary and justifiable.

Treatment options for PNS along the sacral nerve are not well established. Sacrectomy is considered to be the most definitive approach [13] but is highly invasive and often infeasible for levels above S1 [14]. Chemotherapy offers some survival benefits but rarely leads to a cure for stage IV colon cancer, with 5-year survival rates of under 10% [15]. If PNS is considered distant metastasis, it would be considered the same as Stage IV colon cancer, and therefore chemotherapy alone would be expected to be ineffective. Conventional photon radiotherapy has limited efficacy due to the low radiosensitivity of colon cancer [16]. However, recent studies have reported relatively favorable outcomes with high-dose particle beam therapy for presacral recurrence [17]. Given the need for high-dose administration for localized PNS of colorectal cancer, particle beam therapy or high-precision photon radiotherapy like SBRT may be a promising option. More studies, including treatment methods, are needed to examine appropriate management for this condition.

## Conclusions

This report documents a rare case of postoperative PNS in colon cancer that was managed successfully with PBT. The case demonstrates that PBT emerges as a promising local treatment option even in instances of inoperable PNS. PBT is particularly suitable due to its ability to minimize impact on surrounding normal tissues for diseases such as colorectal cancer that necessitate high-dose for local control, as well as in cases where re-irradiation is required. Further evaluation of this treatment will require the collection of more cases treated with PBT under similar conditions.

## References

[1] van den Brink M, Stiggelbout AM, van den Hout WB (2004). Clinical nature and prognosis of locally recurrent rectal cancer after total mesorectal excision with or without preoperative radiotherapy. J Clin Oncol.

[2] Roels S, Duthoy W, Haustermans K, Penninckx F, Vandecaveye V, Boterberg T, De Neve W (2006). Definition and delineation of the clinical target volume for rectal cancer. Int J Radiat Oncol Biol Phys.

[3] Tang Z, Liu L, Liu D (2020). Clinical outcomes and safety of different treatment modes for local recurrence of rectal cancer. Cancer Manag Res.

[4] Takiyama H, Yamada S, Isozaki T, Ikawa H, Shinoto M, Imai R, Koto M (2024). Carbon-ion radiation therapy for unresectable locally recurrent colorectal cancer: A promising curative treatment for both radiation therapy-naïve cases and reirradiation cases. Int J Radiat Oncol Biol Phys.

[5] Capek S, Howe BM, Amrami KK, Spinner RJ (2015). Perineural spread of pelvic malignancies to the lumbosacral plexus and beyond: clinical and imaging patterns. Neurosurg Focus.

[6] (2017). TNM Classification of Malignant Tumours, 8th Edition.

[7] Hiroshima Y, Ishikawa H, Murakami M (2021). Proton beam therapy for local recurrence of rectal cancer. Anticancer Res.

[8] Yi SK, Mak W, Yang CC (2012). Development of a standardized method for contouring the lumbosacral plexus: a preliminary dosimetric analysis of this organ at risk among 15 patients treated with intensity-modulated radiotherapy for lower gastrointestinal cancers and the incidence of radiation-induced lumbosacral plexopathy. Int J Radiat Oncol Biol Phys.

[9] Tunio M, Al Asiri M, Bayoumi Y, Abdullah O Balbaid A, AlHameed M, Gabriela SL, Amir O Ali A (2015). Lumbosacral plexus delineation, dose distribution, and its correlation with radiation-induced lumbosacral plexopathy in cervical cancer patients. Onco Targets Ther.

[10] Hwang ET, Son HM, Kim JY, Moon SM, Lee HS (2020). MR imaging of radiation-induced lumbosacral plexopathy, as a rare complication of concomitant chemo-radiation for cervical cancer. Investig Magn Reson Imaging.

[11] Lee BC, Kim SW, Kim DH, Yoon YC, Kim CK, Sung DH (2022). Lumbosacral plexopathy caused by the perineural spread of pelvic malignancies: clinical aspects and imaging patterns. Acta Neurochir (Wien).

[12] Nathani D, Spies J, Barnett MH, Pollard J, Wang MX, Sommer C, Kiernan MC (2021). Nerve biopsy: current indications and decision tools. Muscle Nerve.

[13] Wanebo HJ, Koness RJ, Vezeridis MP, Cohen SI, Wrobleski DE (1994). Pelvic resection of recurrent rectal cancer. Ann Surg.

[14] Sasikumar A, Bhan C, Jenkins JT, Antoniou A, Murphy J (2017). Systematic review of pelvic exenteration with en bloc sacrectomy for recurrent rectal adenocarcinoma: R0 resection predicts disease-free survival. Dis Colon Rectum.

[15] Loupakis F, Cremolini C, Masi G (2014). Initial therapy with FOLFOXIRI and bevacizumab for metastatic colorectal cancer. N Engl J Med.

[16] Lybeert ML, Martijn H, de Neve W, Crommelin MA, Ribot JG (1992). Radiotherapy for locoregional relapses of rectal carcinoma after initial radical surgery: definite but limited influence on relapse-free survival and survival. Int J Radiat Oncol Biol Phys.

[17] Murayama S, Yamada S, Hiroshima Y (2023). Particle beam therapy for pelvic recurrence of colorectal cancer: a registry data analysis in Japan and a systematic review. J Radiat Res.

